# De Novo Genome Assembly of the Whitespot Parrotfish (*Scarus forsteni*): A Valuable Scaridae Genomic Resource

**DOI:** 10.3390/genes15020249

**Published:** 2024-02-17

**Authors:** Yu Liang, Lin Xian, Jinmin Pan, Kecheng Zhu, Huayang Guo, Baosuo Liu, Nan Zhang, Yan Ou-Yang, Qin Zhang, Dianchang Zhang

**Affiliations:** 1 Guangxi Marine Microbial Resources Industrialization Engineering Technology Research Center, Guangxi Key Laboratory for Polysaccharide Materials and Modifications, School of Marine Sciences and Biotechnology, Guangxi Minzu University, 158 University Road, Nanning 530008, China; 2 Chinese Academy of Fishery Sciences, Key Laboratory of South China Sea Fishery Resources Exploitation and Utilization, Ministry of Agriculture and Rural Affairs, South China Sea Fisheries Research Institute, Chinese Academy of Fishery Sciences, Guangzhou 510300, China; 3 Sanya Tropical Fisheries Research Institute, Sanya 572018, China; 4 Guangdong Provincial Engineer Technology Research Center of Marine Biological Seed Industry, Guangzhou 510300, China

**Keywords:** *Scarus forsteni*, de novo genome assembly, comparative genomics, adaptive evolution, positive genes

## Abstract

*Scarus forsteni*, a whitespot parrotfish from the Scaridae family, is a herbivorous fish inhabiting coral reef ecosystems. The deterioration of coral reefs has highly affected the habitats of the parrotfish. The decline in genetic diversity of parrotfish emphasizes the critical importance of conserving their genetic variability to ensure the resilience and sustainability of marine ecosystems for future generations. In this study, a genome of *S. forsteni* was assembled de novo through using Illumina and Nanopore sequencing. The 1.71-Gb genome of *S. forsteni*, was assembled into 544 contigs (assembly level: contig). It exhibited an N50 length of 17.97 Mb and a GC content percentage of 39.32%. Our BUSCO analysis revealed that the complete protein of the *S. forsteni* genome had 98.10% integrity. Combined with structure annotation data, 34,140 (74.81%) genes were functionally annotated out of 45,638 predicted protein-coding genes. Upon comparing the genome size and TE content of teleost fishes, a roughly linear relationship was observed between these two parameters. However, TE content is not a decisive factor in determining the genome size of *S. forsteni*. Population history analysis results indicate that *S. forsteni* experienced two major population expansions, both of which occurred before the last interglacial period. In addition, through a comparative genomic analysis of the evolutionary relationship of other species, it was found that *S. forsteni* had the closest relationship with *Cheilinus undulatus*, another member of the Labridae family. Our expansion and contraction analysis of the gene family showed that the expansion genes were mainly associated with immune diseases, organismal systems, and cellular processes. At the same time, cell transcription and translation, sex hormone regulation, and other related pathways were also more prominent in the positive selection genes. The genomic sequence of *S. forsteni* offers valuable resources for future investigations on the conservation, evolution, and behavior of fish species.

## 1. Introduction

*Scarus forsteni*, commonly known as the whitespot parrotfish, is a herbivorous species in the family Scaridae, native to tropical areas in the western Pacific Ocean [1]. The high species richness of the Scaridae family, emblematic of reef herbivores, is likely driven by the diversifying pressures of the complex reef ecosystem [2]. The Scaridae family is classified into 10 genera, comprising a total of 90 identified species [3]. Within this family, the genus *Scarus* is the largest one, encompassing about 50 species [4]. Scaridae originated in the late Miocene, around 10 million years ago (Mya), and subsequently underwent a rapid evolutionary split into three major clades [5,6,7]. This diversification continued at a swift pace during the Pleistocene [8,9]. This species diversity, resulting from rapid differentiation, has made parrotfish famous for their bright colors, unique oral morphology, and critical role in coral reef ecosystems. The moniker “parrotfish” is derived from their unique beak-like structure, reminiscent of a parrot’s beak, which is robust and efficacious enough to grind hard coral and rock in their quest for sustenance [10]. Research has indicated that parrotfishes play a role in excavating or scraping algae from coral substrates. Corals and algae share similar ecological niches, and the excavation of algae can increase corals’ light exposure [11]. The ingested material is processed in the pharynx, where three dentate pharyngeal bones, located at the back of the throat, work in conjunction with a special type of monophyodont dentine unique to parrotfish (characterized by pulp filled with alveolar bone), effectively grinding the food into a fine paste [12,13]. This mechanical breakdown promotes the digestion of algal nutrients. It is worth noting that parrotfishes lack a stomach, necessitating the thorough mastication of all ingested substances. After this, fine particles of food are more efficiently decomposed by digestive enzymes. This results in more nutrients being absorbed through the intestinal mucosa, while unabsorbed coral fragments are expelled in the form of white calcium particles [12,14].

Parrotfish, similar to most tropical reef fishes, exhibit hermaphroditism. Typically, hermaphroditic sexual transitions in fish involve substantial and often irreversible alterations in gonadal anatomy and function, accompanied by changes in coloration and behavior. In the case of parrotfish, there exist two or more male morphotypes exhibiting distinct reproductive behaviors, often correlated with variations in gonadal structures and external body morphology [15,16]. Research suggests that this unique reproductive trait may be linked to the coral reefs in which parrotfishes live. They often form small groups in coral reef areas, and some species exhibit territorial behaviors [17,18]. An expanding corpus of research indicates that habitats exert a substantial influence on speciation patterns via sexual selection. In this context, coral reef environments are likely to offer enhanced opportunities for territorial and mating behaviors, thereby intensifying the process of sexual selection [2]. As we all know, parrotfish play a critical role in the health of coral reef ecosystems, especially in the context of current ocean acidification [19]. By grazing on algae that vie for space with corals and by clearing away the overgrowth, they create the open areas essential for coral growth. Additionally, their waste, rich in nutrients, helps sustain the healthy development of corals [11]. The well-being of live corals, serving as a haven for marine fish, is crucial for preserving biodiversity and ensuring ecosystem stability [18,20].

Parrotfish face a significant challenge: they cannot be bred through aquaculture, making their population in the wild particularly precious. Unfortunately, due to their vibrant colors and unique taste, parrotfish have become a high-value target for commercial fisheries. Overfishing, especially for consumption and ornamental purposes, poses a severe threat to these valuable fish species. Parrotfish populations are currently facing significant threats due to overfishing and coral reef degradation, which could also lead to a decline in the genetic diversity of some parrotfish [21,22]. The conservation of these fish and their habitats is imperative for the preservation of the equilibrium and biodiversity within coral reef ecosystems [18,23]. Owing to their distinct biological characteristics and vital roles in ecological processes, parrotfish have emerged as critical subjects in marine biology and environmental protection research [24]. The comprehensive data provided by whole genome sequencing significantly enhances genetic improvement programs across various aquaculture species. Assembling a de novo whole genome is particularly effective in addressing genetic and evolutionary knowledge gaps in non-model organisms [25,26]. Particularly for the genus *Scarus*, this study presents the first key reference genome within the Scaridae family. This reference genome will serve as an invaluable tool for the discovery and genotyping of Single-Nucleotide Polymorphisms (SNPs) from RAD-seq data in closely related species [27,28] in population genomics approaches. In this context, genomic research on parrotfish enhances the conservation of genetic diversity, facilitates the identification of functional genes, and provides insights into evolutionary trends [9,24]. Additionally, previous studies on parrotfish have predominantly focused on mitochondrial genome analysis, population distribution, regional hybridization, and otolith characteristics, with a notable absence of comprehensive genome-wide analysis data [5,7,18,29,30]. The development of new genomic resources is crucial for addressing the current limitations in genetic research on the *S. forsteni* species. High-throughput whole genome sequencing allows us to accurately map the basic genetic blueprint of the species, unlocking the secrets held in its DNA.

In this study, our primary objective was to assemble the genome of the whitespot parrotfish, *S. forsteni*, de novo using both Illumina and Nanopore technologies (i.e., short- and long-read sequencing). Our aim was to generate good-quality data for assembly and annotation to enhance the understanding of the genetic, ecological, evolutionary, and developmental aspects of marine fish, particularly parrotfish. We sought to investigate the relationship between transposable element (TE) content and genome size in parrotfish and its evolutionary significance. Additionally, we aimed to utilize a comparative genomic approach to identify conserved homologous genes in parrotfish and conduct enrichment analysis to gain insights into genes and regulatory elements potentially associated with the species’ dietary traits and digestive system. Overall, our research was designed to augment the pool of good-quality marine fish genomic resources, thereby providing invaluable assets for functional genomic studies and conservation efforts related to parrotfish.

## 2. Materials and Methods

### 2.1. Sample Collection and DNA Sequencing

A single adult female *Scarus forsteni* sample, obtained from Xincun Port in Hainan Province, China [31], was selected for genome sequencing and assembly. Muscle tissue was used for genomic DNA (gDNA) extraction, sequencing, and library construction. High-quality DNA was extracted from fresh muscle tissues using the DNeasy Blood & Tissue Kits (Qiagen, Halden, Germany). DNA quality and quantity were evaluated using electrophoresis on a 1% agarose gel and using Quant-iT™ PicoGreen^®^ dsDNA Reagent and Kits (Thermo Fisher Scientific, Waltham, MA, USA). Then, the DNA was sequenced using two libraries with short and long reads. The library of short sequences was constructed by fragmenting DNA into 300–500 bp fragments using the Covaris 2000 ultrasonicator, followed by purification, end repair, and PCR amplification. This process led to the construction of a DNA library, which was then sequenced on the HiSeq 2500 system with 250 bp PE mode or 100 bp PE mode (Illumina Inc., San Diego, CA, USA). Long-read sequencing involved processing using the Blue Pippin System from Sage Science, USA, to select long fragments targeting a size of approximately 20 kb in preparation for Nanopore sequencing. These fragments were prepared using the 1D Ligation Sequencing Kit (SQK-LSK109) protocol developed by Nanopore, Oxford, UK, with sequencing adapters attached. The genomic DNA libraries, about 20 kb in size, were quantified with Invitrogen’s Qubit 3.0 Fluorometer, Camarillo, USA. The libraries were sequenced on a single flow cell of the PromethION DNA sequencer (Oxford Nanopore, UK) following the manufacturer’s guidelines.

### 2.2. Sequence Data Processing and Genome Survey

The quality of the raw genomic library data from Illumina was assessed using FastQC v.0.11.9 [32,33]. Then, SOAPnuke v2.X [34] was used to exclude low-quality reads after trimming, with low-quality reads being defined by the default parameter (parameters: -l 5 -q 0.3 -n 0.05 -E 50). The trimmed reads processed by SOAPnuke v2.X were utilized for genome size estimation using GCE v1.0.2 software [35]. The frequencies of 17 *k*-mers were counted using GCE v1.0.2 [35] (https://github.com/fanagislab/GCE/tree/master/gce-alternative, accessed on 20 February 2023.), and the heterozygosity and repetitiveness rates of the *S. forsteni* genome were also predicted. The genome size was calculated using the following: size = *k*-mer number/peak depth. Heterozygosity and repetitiveness were calculated using the built-in scripts of the software program.

### 2.3. De Novo Genome Assembly and Quality Assessment

Long-read sequencing was carried out for *S. forsteni* genome assembly, and errors in Nanopore clean reads were corrected using NextDenovo (https://github.com/Nextomics/NextDenovo, accessed on 15 March 2023). The seed threshold was set to 30 k, and the assembly was carried out using the default parameters of the software. Thereafter, the genome assembled via Nanopore technology was refined through two stages of correction using NextPolish [36], which involved the application of both error-corrected long reads and Illumina DNA short reads. To assess the percentage of the original reads represented in the genome assembly, the quality of the *S. forsteni* assembly was evaluated by mapping Illumina reads back to the assembly using BWA-MEM v2 [37]. Finally, the integrity of the genome was assessed using Benchmarking Universal Single-Copy Orthologs (BUSCO) v5.4.4 [38], evaluating 3640 core vertebrate genes with the “actinopterygii_odb10” dataset for 26 fish species (options -m genome).

### 2.4. Gene Prediction and Annotation

Prior to genome annotation, a repeat sequence library was constructed using RepeatModeler (v 2.0.3) [39]. This library was then searched with RepeatMasker (v 4.1.2) [40] to predict repeat sequences in the *S. forsteni* genome. Following this, any overlapping or redundant elements in the predictions from various methods were eliminated. This streamlined process was then used to pinpoint and mask the repetitive sequences in the genome. The repeat-masked genome sequence was then used for subsequent gene annotation. In this study, three strategies were employed to predict the protein-coding genes in the assembled genome, with each chosen strategy being designed to complement the others and enhance the overall accuracy and comprehensiveness of our genomic annotation. Initially, the genome was annotated de novo using the self-training mode of the Augustus software (v 3.4.0) [41]. The annotations were then processed using the SNAP_to_GFF3.pl and augustus_GTF_to_EVM_GFF3.pl scripts from the EvidenceModeler (EVM, v 1.1.1) [42] package. Second, protein-coding genes were predicted based on transcriptome data using the TransDecoder-v5.7.0 software (https://github.com/TransDecoder/TransDecoder, accessed on 25 April 2023), and RNA sequencing (RNA-seq) data were obtained from the unpublished data of our research group. Third, protein sequences of *Labrus bergylta*, *Cheilinus undulatus*, *Notolabrus celidotus*, *Sparus aurata*, and *Acanthopagrus latus* were downloaded from the NCBI database (https://www.ncbi.nlm.nih.gov, accessed on 25 April 2023) for homology prediction with the parrotfish genome. Genewise [43] was then utilized to analyze these alignments, aiming to accurately determine spliced alignments. Finally, EVM was used to integrate the protein-coding gene predictions to form a consensus gene set.

The functional annotation of predicted protein-coding genes was performed using BLAST (blastp) with an e-value of 1 × 10^−5^ against several databases [44]: the SWISS-PROT database [45], the NCBI non-redundant protein (NR) database, the Translated EMBL (TrEMBL) database, the Clusters of Orthologous Genes (COG) database, and the Kyoto Encyclopedia of Genes and Genomes (KEGG) database [46]. Additionally, protein motifs and domains were annotated by searching the InterPro and Gene Ontology (GO) databases using InterProScan (v.4.8) [47]. Infernal v1.1.2 software was used to predict partial non-coding proteins [48].

### 2.5. Repetitive Element Annotation

In teleost genomes, transposable elements (TEs) were identified through a combination of homology-based and de novo approaches. Known TEs were screened using RepeatMasker (v 4.1.2) and RepeatProteinMask against the RepBase library (http://www.repeatmasker.org/, accessed on 25 April 2023) for homology-based detection [40]. Additionally, de novo TEs were pinpointed by RepeatMasker (v 4.1.2), utilizing a library constructed using RepeatModeller2 and LTR_FINDER (v.1.0.5) [39,49]. Furthermore, Tandem Repeats Finder (TRF, v.4.07b) [50] was employed to detect tandem repeats under default settings. This study also compared the content of transposable elements in *Amphiprion ocellaris*, *Oreochromis niloticus*, *Oryzias latipes*, *Larimichthys crocea*, *Chelmon rostratus*, *Danio rerio*, and *S. forsteni* [51,52,53,54,55].

### 2.6. Population History Analysis

To gain a deeper understanding of the population dynamics and history of *S. forsteni*, we employed the Pairwise Sequentially Markovian Coalescent (PSMC v0.6.5) model to infer its population size history from a diploid sequence [56]. This analysis helped reveal the species’ adaptations and evolutionary processes in response to past environmental changes and ecological pressures, providing valuable insights into its population history. To generate the whole genome diploid consensus sequence, clean data were mapped to the *S. forsteni* assembled genome using the BWA-MEM v2 [37]. SAMtools v1.13 [57] was then employed to produce the diploid consensus under default parameters (https://github.com/lh3/psmc, accessed on 25 October 2023), with the parameters set to “-d 10 -D 100”. PSMC model estimates were run with the options -N25 -t15 -r5 -p “4 + 25 × 2 + 4 + 6”, assuming a generation time of 2 years for *S. forsteni* and a substitution rate of 2.0 × 10^−9^ per site per year [56,58].

### 2.7. Phylogenetic Analysis and Divergence Time Estimation

To determine the phylogenetic position of *S. forsteni*, phylogenetic analysis was conducted using single-copy homologous genes from its genome and those of thirteen teleost fishes (*O. niloticus*, *O. latipes*, *L. crocea*, *C. rostratus*, *Plectropomus leopardus*, *Archocentrus centrarchus*, *Labrus bergylta*, *C. undulatus*, *Notolabrus celidotus*, *D. rerio*, *Acanthochromis polyacanthus*, *A. ocellaris*, *Stegastes partitus*) and one outgroup (*Homo sapiens*). The genomic data for these species, obtained from NCBI, were analyzed for homologous clustering using the OrthoFinder v2.5.4 [59]. Subsequently, based on the above results, the unique genes of *S. forsteni* were identified, and the single-copy homologous genes of each species were aligned using MAFFT v7.490 [60] to create a super-alignment matrix. A phylogenetic tree was then constructed using RAXML v8.2.12 [61] with default settings. In this study, divergence times for 14 species were estimated using a phylogenetic tree with specific time nodes using the Mcmctree program from PAML v4.9. This involved five calibration points: the divergence between *Danio rerio* and *Homo sapiens* (approx. 423.3–440.0 Mya), *D. rerio* and *Sparisoma partitus* (approx. 180.0–251.5 Mya), *S. partitus* and *Ambloplites centrarchus* (approx. 74.4–96.5 Mya), *Labrus bergylta* and *Cichla undulatus* (approx. 51.8–63.1 Mya), and *Cichla rostratus* and *N. celidotus* (approx. 83.2–142.9 Mya) [62]. Additionally, we conducted statistical comparisons of multi-copy orthologs, unique paralogs, other orthologs, and unclustered genes in the predicted gene libraries, across the species.

### 2.8. Expansion and Contraction of Gene Families

Gene family expansions, involving increased gene copies, and contractions, with reduced gene copies, were central to our study. We conducted an expansion and contraction analysis of single-copy homologous gene families across 15 species to understand their evolutionary dynamics and functional implications. Our main goal was to explore how gene families evolved within these species and uncover potential adaptations or functional changes in an evolutionary context. Gene family expansions and contractions were examined using CAFÉ v5.0 [63] based on orthologs identified from gene family clustering and a phylogenetic analysis of single-copy orthologous genes. Variations in gene families along each lineage of the phylogenetic tree were predicted using a random birth and death model. The significance of gene family changes was assessed by comparing conditional likelihoods from a probabilistic graphical model using *p* values. GO term and KEGG pathway enrichment analyses were conducted based on gene families specifically expanded (*p* < 0.05) in *S. forsteni* using clusterProfiler [64].

### 2.9. Screening of Positive Selection Genes

To elucidate the mechanisms underlying the adaptive evolution of *S. forsteni* and to identify specific genes that may have played a crucial role in its unique adaptations, we conducted a detailed analysis of selective pressures using single-copy orthologous genes from 15 species. The ratio of non-synonymous substitution (Ka) to synonymous substitution (Ks), namely the ω value (Ka/Ks), is commonly employed to represent the selection pressure in sequence analysis. In this research, PAML v.4.9 software [62] and its CODEML tool were used to estimate the Ka/Ks (omega) values. The branch-site model was chosen due to its better alignment with the actual scenarios of intricate species divergence processes. For each gene, CODEML was used to calculate the likelihood value [65], and this value was further utilized to compute the likelihood ratio statistic. Subsequently, a chi-square test was applied to obtain the corresponding p-value. To control for false discovery rate (FDR) across the entire genome, all *p*-values were adjusted accordingly. Genes with FDR values less than 0.05 were selected as the final-candidate positively selected genes [66]. Next, eggnog-mapper v2.1 [67] was used to further annotate the resulting positive selection genes. Finally, using clusterProfiler [64], GO term and KEGG pathway enrichment analyses were performed on gene families that showed positive selection (*p* < 0.05) in *S. forsteni*.

## 3. Results

### 3.1. Genome Assembly and Quality Assessment

**Figure 1:**
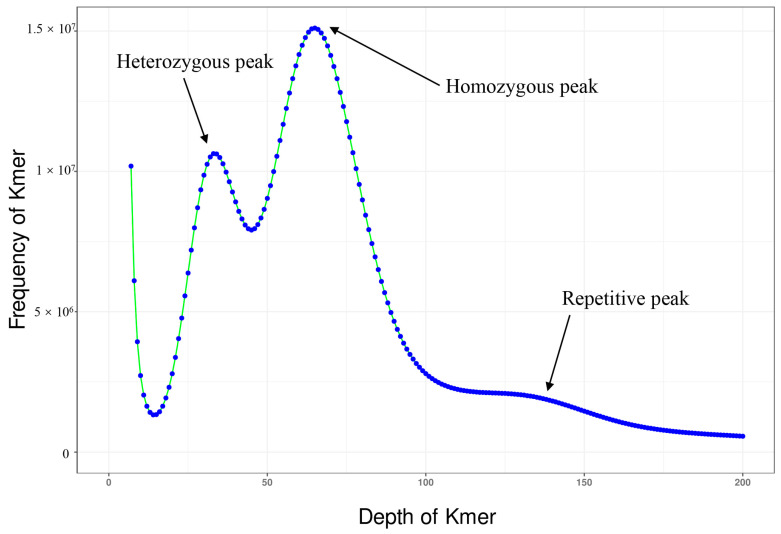
Seventeen- *k*-mer depth distribution of the *Scarus forsteni* genome.

Our sequencing project generated approximately 123.04 Gb of raw Illumina paired-end reads. These were processed using SOAPnuke v2.X software to remove low-quality reads and adapter sequences, resulting in ~109 Gb of clean data. This high-quality dataset was then utilized for genome size estimation. The GCE v1.0.2 was utilized to generate a histogram for sequencing depth distribution (*k* = 17) (Figure 1). Upon the visualization of our results, a single *k*-mer coverage peak at approximately 65× coverage depth, indicative of a homozygous peak, was revealed. Additionally, a heterozygous peak at around 30× coverage depth and a peak corresponding to repetitive sequences between 100× and 150× coverage depth were also observed. As estimated by the GCE v1.0.2 software, the genome size was approximately 1.61 Gb. A script-based calculation revealed a heterozygosity rate of 0.78%, classifying it as a genome with low heterozygosity. Utilizing Oxford Nanopore sequencing technology, we obtained 169 GB of raw data. These raw data were then subjected to genome assembly using NextDenovo software V2.1-beta.0 with the default parameters set for raw data input. Subsequently, adapter sequences were removed and errors corrected using NextPolish, resulting in an optimized draft genome of the parrotfish (assembly level: contig) with a total size of 1.71 Gb. The assembly comprised 544 contigs, with the largest contig being 58.57 Mb. The N50 length was 17.97 Mb, and the GC content was 39.32% (Table 1). This species’ genome is larger than the known genomes of other marine fishes. Searches for BUSCO v5.4.4 using the actinopterygii_odb10 core gene sets showed that the assembly genome contained 98.10% of the complete sequences and 0.58% of the fragmented sequences of genes, with 1.32% of the genes being missing (Table 2). Moreover, 1.57% of the genes were identified as duplicates. Upon comparison with Illumina DNA short reads, facilitated by using BWA-MEM v2 software, it was found that 95.87% of the reads were correctly matched. These results indicated that our genome had a high assembly efficiency and integrity.

### 3.2. Genome Annotation

**Figure 2:**
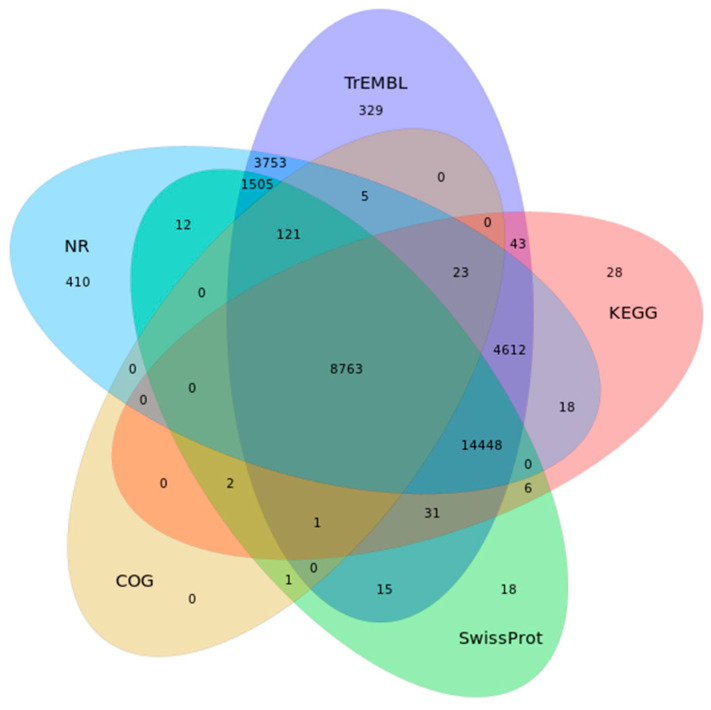
Venn diagram showing the *Scarus forsteni* genome in five functional annotation databases of common and unique genes.

De novo and homologous annotations were used to predict gene structure, together with transcriptome data, resulting in 45,638 predicted genes for gene annotation. Using BLAST (blastp) for functional annotation against public databases such as COG, KEGG, NR, SWISS-PROT, and TREMBL, 34,140 of the predicted protein-coding genes were functional, accounting for 74.81% of the predicted genes. Among them, 54.61% of the genes were compared with SWISS-PROT, and more than 70% of the genes were annotated in NR and TREMBL. And a total of 8763 genes could be annotated in all databases (Figure 2, Table S1). In addition, our genomic analysis predicted the presence of non-protein-coding genes, namely 251 rRNA genes, 2028 tRNA genes, 473 small-nuclear RNA (snRNA) genes, and 350 microRNA (miRNA) genes (Table 3).

### 3.3. Repetitive Element Annotation

**Figure 3:**
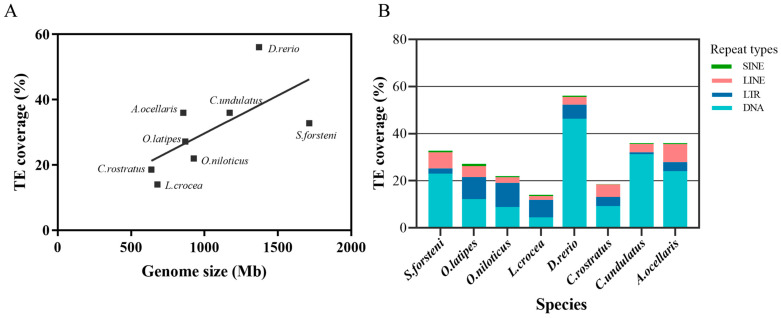
(**A**) Correlation between genome size and transposable element content in teleosts. (**B**) Total amount and relative proportions of DNA transposons, LTRs (Long Terminal Repeats), LINEs, and SINE (Short Interspersed Nuclear Element) retrotransposons in teleost genomes.

Repeat sequence elements of the *S. forsteni* genome were annotated by using RepeatMasker (v 4.1.2) and RepeatModeller2. The sequence of repetitive elements contributed 880.8 Mb of the assembly and accounted for ~51.4% of the genome length (Table 4). In the *S. forsteni* genome assembly, it was observed that class I TEs (RNA transposons or retrotransposons) comprised approximately 9.74% of the genome. The predominant retrotransposons identified were Long Interspersed Nuclear Elements (LINEs), accounting for 70.63% of all class I transposons detected. Additionally, the genome of *S. forsteni* was found to be abundant in class II TEs (DNA transposons), constituting nearly 23.04% of the total genome. In a comparative analysis of TE content across *S. forsteni* and seven other species, a positive correlation regarding the relationship between TE content and genome size was observed, and this correlation is approximately linear (*p* value: 0.0825) (Figure 3A, Table S2). For other fish species that evolved later, such as *S. forsteni*, *C. undulatus*, and *A. ocellaris*, the content of Transposable Elements (TEs) was found to be similar (Figure 3B).

### 3.4. Population History of S. forsteni

**Figure 4:**
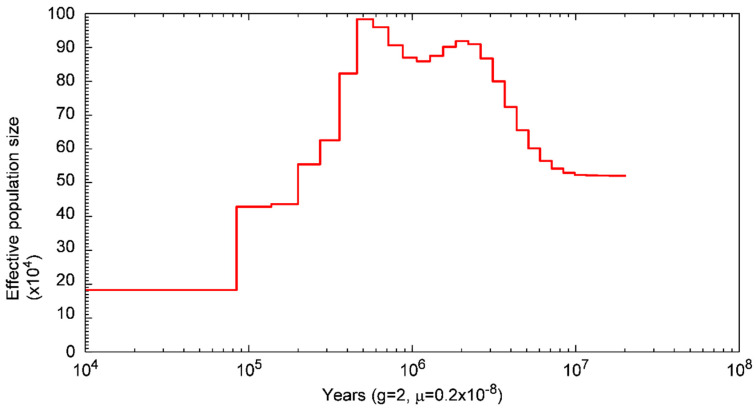
Fluctuation in population size of *Scarus forsteni* between 100 Ma and 10 Ka (g: generational time in years, µ: every-generation mutation rate).

In order to gain insights into the historical population dynamics of *S. forsteni*, this study employed a Pairwise Sequentially Markovian Coalescent (PSMC) analysis. This method infers past effective population sizes over different time points by assessing the distribution of coalescent times across various segments of a single individual’s genome sequence. PSMC analysis is widely regarded as an effective tool for studying population history and dynamics, being particularly suitable for species where extensive historical samples are not readily available. The effective population size of *S. forsteni* between 10 Ka and 100 Ma is shown in Figure 4. The visualizations show that between 10 Ma and 4 Ma, there was a marked expansion in population size, reaching a peak at 4 Ma. This was followed by a slight decline in population size, which then began to expand again around 1000 ka, reaching a second peak, and became the highest at 700 Ka. Subsequently, the population size showed a stepwise decline until 100 Ka.

### 3.5. Genome Evolution Analysis

**Figure 5:**
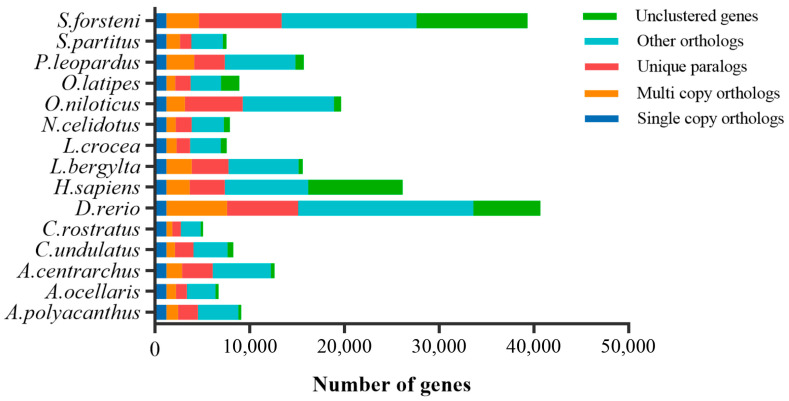
The protein-coding genes of the total 15 species were clustered into 3234 gene families. Among these gene families, 1196 were single-copy gene families (one copy in each of these species).

**Figure 6:**
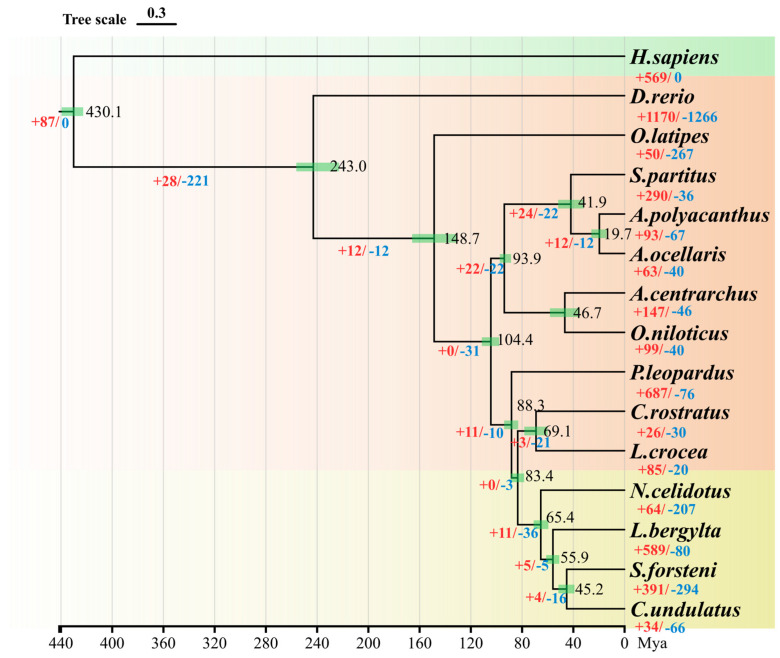
Phylogeny construction based on the single-copy gene families. The black number represents the node differentiation time, the red number represents the expansion gene family number, and the blue number represents the contraction gene family number. The green layer represents outgroups, the orange layer represents other teleosts, and the yellow layer represents the Labridae family.

To investigate the phylogenetic relationship of the *S. forsteni* with 14 species—*O. niloticus*, *O. latipes*, *L. crocea*, *C. rostratus*, *P. leopardus*, *A. centrarchus*, *L. bergylta*, *C. undulatus*, *N. celidotus*, *D. rerio*, *A. polyacanthus*, *A. ocellaris*, *S. partitus*, and *H. sapiens*—we conducted a comparative genomics analysis. The results show that the protein-coding genes of the total 15 species were clustered into 3234 gene families. Among these gene families, 1196 were single-copy gene families (Figure 5), and 42,555 genes from 30,862 orthogroups were identified in S. forsteni. Upon analyzing the data for single-copy orthologs, multi-copy orthologs, unique paralogs, other orthologs, and unclustered genes across the 15 species, it was discovered that *S. forsteni*, similar to both zebrafish and humans, possesses a significantly high number of genes. Next, we utilized the coding sequences of 1196 single-copy homologous genes to construct a phylogenetic tree and determine divergence times (Figure 6). According to our phylogenetic analysis, *S. forsteni* and *C. undulatus*, as well as *N. celidotus* and *L. bergylta*, were members of the Labridae family. It was determined that *S. forsteni* and *C. undulatus* diverged from a common ancestor approximately 45.2 Mya.

### 3.6. Expansion and Contraction of Gene Families

**Figure 7:**
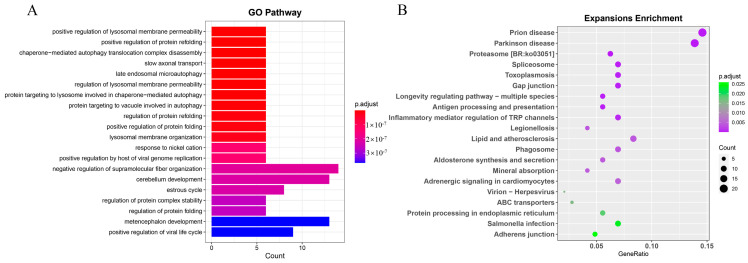
GO term and KEGG enrichment analyses of *Scarus forsteni* expansion gene families. (**A**) GO term. (**B**) KEGG.

**Figure 8:**
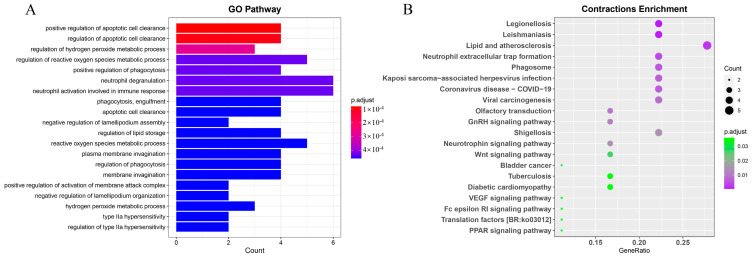
GO term and KEGG enrichment analyses of *Scarus forsteni* contraction gene families. (**A**) GO term. (**B**) KEGG.

To investigate the adaptive evolution of *S. forsteni*, we estimated the expansion and contraction of the gene families. A total of 391 genes were identified as expanded in the *S. forsteni* genome, and 294 were identified as contracted in the *S. forsteni* genome. GO term and KEGG pathway enrichment analyses were performed for 197 significantly expanded genes (*p* < 0.05) (Tables S3 and S4) and 19 significantly contracted genes (*p* < 0.05) (Tables S5 and S6). The top 20 pathways from the KEGG enrichment analysis revealed that the expanded genes were primarily enriched in categories such as human diseases, organismal systems, and cellular processes. Specifically, this was manifested in pathways associated with prion disease, Parkinson’s disease, lipid and atherosclerosis, immune system, gap junction, mineral absorption, and phagosomes. Our GO term analysis of the expanded genes showed a higher number of genes in pathways related to processes such as the negative regulation of supramolecular fiber organization, cerebellum development, and metencephalon development (Figure 7). In addition, the enrichment of the contracted gene families was mainly demonstrated in olfactory transduction, the neurotrophin signaling pathway, and the regulation of apoptotic cell clearance (Figure 8).

### 3.7. Analysis of Positive Selection Genes 

**Figure 9:**
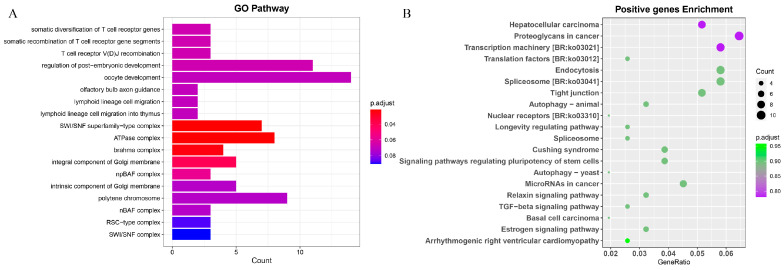
GO term and KEGG enrichment analyses of *Scarus forsteni* positive selection genes. (**A**) GO term. (**B**) KEGG.

According to the branch-site of *S. forsteni* in the phylogenetic tree, we identified that 206 genes were subjected to significantly positive selection (*p* < 0.05). Our KEGG enrichment analysis indicated significant involvement in Brite Hierarchies such as transcription machinery, translation factors, and the spliceosome. Additionally, there was notable representation in the signaling pathways regulating the pluripotency of stem cells, tight junctions, and the estrogen signaling pathway. The regulation of post-embryonic development, oocyte development, polytene chromosome development, and various complexes were predominantly featured in the GO term analysis (Figure 9; Tables S7 and S8).

## 4. Discussion

### 4.1. Genome Assembly and Annotation

In this study, the size of the genome of whitespot parrotfish was estimated to be about 1.61 Gb, according to our *k*-mer analysis. This analysis not only provided an estimate of genome size but also revealed insights into the genome’s complexity, repetitive elements, error rates, polymorphism levels, and correlation with GC content [35]. However, based on de novo assembly, we obtained a genome size of 1.71 Gb for the whitespot parrotfish. This variation in genome size may be due to changes in the quality and depth of sequencing data or assembly strategies [68,69]. Among them, we found that the whitespot parrotfish exhibited higher heterozygosity compared to *C. undulatus* [70]. Our *k*-mer analysis revealed the presence of highly repetitive and heterozygous regions in the parrotfish genome. This may explain why our genome size result obtained from de novo assembly (1.71 Gb) was larger than that obtained from the *k*-mer analysis. These features may have implications for the actual genome assembly process [71]. The GC content is an important sequence index affecting the randomness of genome sequencing [72]. It was revealed that GC content of whitespot parrotfish is 39.32%, which is similar to that of species with relatively smaller genome sizes, such as *Symphodus melops* (41.8%), *Semicossyphus pulcher* (41.65%), *Labroides dimidiatus* (40.8%), and *Labrus mixtus* (41%, https://www.ncbi.nlm.nih.gov/datasets/genome/GCF_963584025.1/, accessed on 25 October 2023) [73,74,75]. In certain fish species, a negative correlation has been observed between GC content and genome size, indicating that species with larger genomes tend to have lower GC contents [70,76]. However, this trend was not universally observed across all species. Moreover, N50 mainly measures the average length of the assembly sequence, and a higher N50 value usually means that the assembly contains a longer sequence, which can be seen as a sign of good assembly quality [77]. The whitespot parrotfish genome had an N50 length of 17.97 Mb, indicating a good-quality assembly. BUSCO estimated the completeness and redundancy of processed genomic data based on universal single-copy orthologs [38]. In the BUSCO analysis, a comparison against the Actinopterygii_odb10 core gene set revealed that 3571 out of 3640 genes were present in the *S. forsteni* genome, achieving a completeness of 98.10%. This was a relatively good result for a contig-level genome. This was slightly higher than the 96.36% completeness observed in *C. undulatus* [70]. Additionally, comparing annotation statistics from genomes closely related to our subject serves as an indirect indicator of the assembly’s quality [26]. Of the predicted 45,638 protein-coding genes, 34,140 coding sequences showed homology to each database protein in functional annotation and were successfully annotated. That is more than the number of proteins encoded by most marine fish [25,70,78]. Overall, the assembly of the *S. forsteni* genome was of good quality, rendering it suitable for subsequent research and in-depth analysis.

### 4.2. Repetitive Element Analysis

Among repetitive elements, transposable elements (TEs) received the most attention. TEs are DNA sequences capable of changing their positions within the genome, sometimes causing or reversing mutations and altering the genetic characteristics and genome size of a species [76,79]. Research has found that TE content varies, ranging from 5% in pufferfish to 56% in zebrafish, and it has been positively correlated with the genome size of fish [55]. Our results also exhibited a similar trend regarding this correlation. The content of repetitive sequences in the genome of *S. forsteni* was excessively high, which may be closely related to its high content of TEs. Class I TEs and Class II TEs account for 9.74% and 23.04% of the genome, respectively. The predominance of Class II TEs in teleost genomes suggests a significant composition of DNA transposons, coupled with a relatively sparse presence of ancient TE copies. This pattern indicates that DNA transposons play a dominant role in shaping the genomic architecture of these species [53]. Some studies have shown that the content of transposons in different species is closely related to their historical evolution and genome size [51,53,80]. Throughout evolutionary history, the size of genomes and the occurrence of transposable elements has affected the structural and functional aspects of both genomes and cells. Variations in these aspects have impacted the morphological and functional features of organisms upon which natural selection exerts its influence directly [81]. In a comparative analysis of TE content across *S. forsteni* and seven other species, a positive correlation was observed, indicating that the relationship between TE content and genome size is approximately linear. As an earlier evolved species, zebrafish, with its larger genome size, encompassed a higher proportion of TEs. In comparison with other later-evolved fish species, the TE content in *S. forsteni*, *C. undulatus*, and *A. ocellaris* was found to be similarly high, exceeding 30% in each of these species (Figure 3, Table S2). Additionally, *S. forsteni* and *C. undulatus*, which are phylogenetically sister taxa, also exhibited relatively larger genome sizes compared to other parrotfish species. In summary, the high content of repetitive sequences, especially TEs, in the *S. forsteni* genome suggests their potential influence on genome size, but this relationship is not conclusive, and additional research is required.

### 4.3. Population History of S. forsteni

Past population sizes can be inferred based on the representative genome sequence of a species. This approach enables the exploration of biological questions such as the impacts of climatic events on population size and structure, the effects of human activities on wildlife populations, and the consequences of domestication [82,83,84,85,86]. The Pairwise Sequentially Markovian Coalescent (PSMC) model is a mature analytical method for inferring population dynamics [56]. Our PSMC analysis of *S. forsteni* showed that the population increased to 4 Ma and then began to decline. This may be because, 2.15 Mya, a large asteroid (more than 1 km in diameter) was thought to have fallen into the Southern Ocean in the Eltanin Fault zone, generating a super-tsunami that resulted in large-scale marine extinction [87]. In addition, affected by the mid-Pleistocene climate transition (MPT, 1.2–0.55 Ma) feature (glacial-interglacial cycle alternating pattern), the population size of the whitespot parrotfish experienced a small expansion followed by a sustained contraction during this period [88,89]. Interglacial global warming and glacier retreat may have contributed to the expansion of the whitespot parrotfish population by creating conditions for the recovery and expansion of coral reefs [90]. The second decline in population size around 700 Ka may be related to the mid-Pleistocene climate transition that occurred approximately 0.9 Ma [89]. Extreme glacial environmental conditions led to a general decline in the abundance and diversity of species prone to extinction. Our findings are similar to those of analyses on the population history of Catfishes conducted by other researchers [91]. In summary, we found that the expansion and contraction of the whitespot parrotfish population all occurred before the last interglacial period [92].

### 4.4. Genome Evolution Analysis

Studies have shown that to best understand the evolution of the forms, functions, and behaviors of different organisms, it is essential to combine the phylogenetic information of a population with mechanistic studies of organismal function. This approach helps to create a comprehensive picture of the historical changes in key features [93]. Scaridae, as one of the coral reef fish families, exhibited exceptional diversity in its body size, shape, coloration, feeding habits, reproductive behaviors, and life histories, all of which may be closely related to its evolutionary adaptations [24,94]. To explore the evolutionary relationship between *S. forsteni* and other vertebrates, a phylogenetic tree was reconstructed. This was based on the analysis of 1196 single-copy orthologs derived from comparative genomics studies of the genomes of 15 species. According to our phylogenetic analysis, *S. forsteni* and *C. undulatus*, as well as *N. celidotus* and *L. bergylta*, had a common ancestor because they all belong to the Labridae family. Compared with other teleosts, they began to differentiate about 65.4 Mya, which led to significant changes in global temperature due to the alternation of glaciation and interglaciation [95]. These climatic fluctuations, particularly during the warmer interglacial periods, led to sea level rises, which may have facilitated the expansion and recovery of coral reef habitats. This expansion of coral reefs likely provided new ecological niches, thereby potentially increasing the diversity of species adapted to these environments. Consequently, it is hypothesized that these environmental changes contributed to the diversification observed within the Labridae family [6,96]. Next, it was found that *S. forsteni* and *C. undulatus* are closely related in phylogeny. In our phylogenetic analysis, at the genomic level, it was estimated that *S. forsteni*, *C. undulatus*, and their common ancestor diverged approximately 45.2 Mya. This is also a plausible time frame for Labridae species to have begun participating in adaptive evolution [8]. However, the karyotypic differences that emerged during the evolutionary process have led to distinctions between the two species, including in morphological and dietary traits [7,70]. *Scarus* species prefer to graze on coral, while *Cheilinus* species are more inclined to feed on sea grass, and *Notolabrus* species primarily target bivalves, crabs, and gastropods [7,97]. Additionally, research has found that the Labridae are traditionally classified in the suborder Labroidei with the families Scaridae, Odacidae, Cichlidae, Pomacentridae, and Embiotocidae [98]. Thus, Scaridae and Pomacentridae are sister groups, and the dichotomous evolutionary network pattern along the ecological morphological axis that they exhibit is similar to the findings of our study [24,94]. The preservation of territorial behavior within their habitats may be attributed to their common evolutionary ancestry [99,100].

### 4.5. Enrichment of Expansion and Contraction Gene Families

Understanding the evolution of fish gene families is of significant importance for the conservation of the genetic resources of fish. Studies have contributed to unveiling the origins and evolutionary processes of biodiversity, consequently providing valuable insights for the development of conservation strategies and the sustainable management of fishery resources [63,70]. In our analysis of the expansion and contraction of the gene families of whitespot parrotfish, the number of expanded gene families exceeded the number of contracted gene families. Through our KEGG enrichment analysis, we found that the expanded genes were primarily enriched in categories such as prion disease, Parkinson’s disease, lipid and atherosclerosis, immune system, gap junction, mineral absorption, and phagosomes. Among them, a large number of enriched genes were found to belong to the disease defense system. As species adapt to new environments, they are constantly exposed to new pathogens and stimuli that cause the immune system to evolve [101,102]. High levels of PUFA were found in *Scarus rivulatus*, which may be attributed to its frequent consumption of unsaturated fat from coral algae, crustaceans, and polychaetes. This diet likely induces the endogenous de novo biosynthesis of fatty acids and elongated chains, contributing to lipid and energy metabolism through the lipid and atherosclerosis pathway [103,104]. At the same time, the mineral absorption pathway may promote the mineral utilization and metabolism of parrotfish for algae and debris scraped off the surface of coral reefs [11,13]. The processing of ABC transporters and proteins in the endoplasmic reticulum, as the most basic biological process in body function, involves the transport of many kinds of molecules. It may be very important to the energy metabolism and excretion process of parrotfish. [105]. In addition, we found that olfactory transduction in parrotfish showed a contractile state during the evolution of parrotfish. This observation becomes particularly interesting when considering the behavior of coral reef fish eggs. After two days of incubation, coral reef fish eggs floated away with the current, and they might have returned to their birth reef by smell when they became capable of swimming [106]. These analyses demonstrated that the gene families of the parrotfish had undergone expansion and contraction during their evolutionary process, significantly influenced by environmental factors. This has enabled the physiological and behavioral traits of the parrotfish to adapt more effectively to ever-changing environmental conditions.

### 4.6. Analysis of Positive Selection Genes

Positive selection analysis was instrumental in identifying genes that had been subjected to natural selection pressures during the evolutionary process. These genes were often associated with improvements in a species’ adaptability to specific environmental conditions, such as resistance to diseases, adaptation to particular ecological niches, or coping with environmental changes [107]. Through the use of the KEGG and GO databases for our enrichment analysis of positively selected genes in parrotfish, it was found that these genes, which encode proteins, are mainly associated with cell transcription and translation, as well as sex hormone regulation. Additionally, there was notable representation in transcription machinery, translation factors, signaling pathways regulating the pluripotency of stem cells, the TGF-β signaling pathway, the estrogen signaling pathway, the regulation of post-embryonic development, and oocyte development. Sexual reversal, including the transformation of primordial females into preferential females, is a common phenomenon observed in most parrotfish species [15]. In certain species, when a group of females lived together within the territory of a single large, brightly colored male, if that male was removed from the group, the largest female in the colony could undergo a process known as sexual reversal. This means that she changes both her coloration and behavior to take on the characteristics of a male [9]. The enrichment of the estrogen signaling pathway, oocyte development, and the regulation of post-embryonic development has been suggested to be closely associated with sex differentiation, sexual maturation, and sexual behavior in parrotfish. This phenomenon has sparked significant interest among researchers, making the mechanisms of sex determination in vertebrates a highly debated and extensively studied topic [52,108,109]. These results indicated that under the influence of natural selection, genes in *S. forsteni* associated with specific traits have evolved adaptively.

## 5. Conclusions

This study marks the first sequencing and analysis of the *S. forsteni* genome, providing a pivotal reference genome for the Scaridae family. Our in-depth analysis of the 1.71 Gb genome revealed high genomic integrity and a wealth of functionally annotated genes. Additionally, through comparative genomic approaches, we have uncovered key genetic factors influencing traits in the Labridae family, as well as enrichment pathways related to immune diseases, organismal systems, cellular processes, and sex reversal. These findings not only offer new insights into the evolutionary history of *S. forsteni* and its relatives but also lay the groundwork for future conservation and management strategies for coral reef fishes. We anticipate that future research will further explore the role of these genomic features in coral reef ecosystems and how they impact the adaptability and survival strategies of coral reef fishes.

## Figures and Tables

**Table 1:** Statistics regarding *Scarus forsteni* genome assembly.

Item	Value
Total length (bp)	1,713,752,192
Total contigs	544
GC (%)	39.32
N50 (bp)	17,972,633
N90 (bp)	1,420,458
Ave length (bp)	3,150,279
Max length (bp)	58,576,118
Min length (bp)	35,884

**Table 2:** BUSCO analysis statistics in the genome of *Scarus forsteni*.

Item	Number	Percent (%)
Complete BUSCO (C)	3571	98.10
Complete and single-copy BUSCO (S)	3514	96.54
Complete and duplicated BUSCO (D)	57	1.57
Fragmented BUSCO (F)	21	0.58
Missing BUSCO (M)	48	1.32
Total BUSCO groups searched	3640	100.00

**Table 3:** Statistics of the predicted non-coding RNAs in the genome of *Scarus forsteni*.

Non-Code RNA Type	Copy Number	Total Length (bp)	Avg Length (bp)
tRNA	2028	151,198	74.5552
rRNA	251	92,899	370.116
snRNA	473	61,713	130.471
miRNA	350	26,745	76.4143

**Table 4:** Repetitive element annotations in the genome of *Scarus forsteni*.

Type of Repeat	Length (bp)	% of Genome
SINEs	11,580,806	0.68
LINEs	117,969,346	6.88
L2/CR1/Rex	77,550,358	4.53
R1/LOA/Jockey	7,296,609	0.43
R2/R4/NeSL	2,468,557	0.14
RTE/Bov-B	10,610,769	0.62
L1/CIN4	13,198,944	0.77
LTR	37,360,206	2.18
BEL/Pao	2,804,533	0.16
Ty1/Copia	152,646	0.01
Gypsy/DIRS1	13,611,972	0.79
Retroviral	6,496,637	0.38
DNA transposons	394,903,094	23.04
hobo-Activator	1,59,106,235	9.28
Tc1-IS630-Pogo	73,869,980	4.31
PiggyBac	6,409,425	0.37
Tourist/Harbinger	39,804,001	2.32
Other	3,827,180	0.22
Rolling-circles	10,770,335	0.63
Unclassified	280,705,119	16.38
Satellite	4,266,447	0.25
Simple-repeat	20,194,587	1.18
Low complexity	2,709,891	0.16
Total	880,459,831	51.38

## Data Availability

The genome data have been stored in GenBank under BioProject number PRJNA1068821.

## Supplementary Materials

The following supporting information can be downloaded at: https://www.mdpi.com/article/10.3390/genes15020249/s1, Table S1: Statistics of the functional annotation of genes. Table S2: Genome size and proportion of DNA transposons, LTRs, LINEs, and SINEs in eight teleosts. Table S3: The KEGG enrichment of expansion gene families in *Scarus forsteni*. Table S4: The GO term of expansion gene families in *S. forsteni*. Table S5: The KEGG enrichment of contraction gene families in *S. forsteni.* Table S6: The GO term of contraction gene families in *S. forsteni*. Table S7: The KEGG enrichment of positive selection genes in *S. forsteni*. Table S8: The GO term of positive selection genes in *S. forsteni*.

## References

[1] Darius H.T., Paillon C., Mou-Tham G., Ung A., Cruchet P., Revel T., Viallon J., Vigliola L., Ponton D., Chinain M. (2022). Evaluating Age and Growth Relationship to Ciguatoxicity in Five Coral Reef Fish Species from French Polynesia. Mar. Drugs.

[2] Kazancioglu E., Near T.J., Hanel R., Wainwright P.C. (2009). Influence of Sexual Selection and Feeding Functional Morphology on Diversification Rate of Parrotfishes (Scaridae). Proc. Biol. Sci..

[3] Fitzpatrick J.M., Carlon D.B., Lippe C., Robertson D.R. (2011). The West Pacific Diversity Hotspot as a Source or Sink for New Species? Population Genetic Insights from the Indo-Pacific Parrotfish *Scarus rubroviolaceus*. Mol. Ecol..

[4] Gomi K., Nakamura Y., Kanda M., Honda K., Nakaoka M., Honma C., Adachi M. (2021). Diel Vertical Movements and Feeding Behaviour of Blue Humphead Parrotfish Scarus Ovifrons in a Temperate Reef of Japan. J. Fish Biol..

[5] Carlon D.B., Robertson D.R., Barron R.L., Choat J.H., Anderson D.J., Schwartz S.A., Sánchez-Ortiz C.A. (2021). The Origin of the Parrotfish Species Scarus Compressus in the Tropical Eastern Pacific: Region-Wide Hybridization between Ancient Species Pairs. BMC Ecol. Evol..

[6] Cowman P.F., Bellwood D.R., van Herwerden L. (2009). Dating the Evolutionary Origins of Wrasse Lineages (Labridae) and the Rise of Trophic Novelty on Coral Reefs. Mol. Phylogenet. Evol..

[7] Gao J., Li C., Yu D., Wang T., Lin L., Xiao Y., Wu P., Liu Y. (2023). Comparative Mitogenome Analyses Uncover Mitogenome Features and Phylogenetic Implications of the Parrotfishes (Perciformes: Scaridae). Biology.

[8] Alfaro M.E., Brock C.D., Banbury B.L., Wainwright P.C. (2009). Does Evolutionary Innovation in Pharyngeal Jaws Lead to Rapid Lineage Diversification in Labrid Fishes?. BMC Evol. Biol..

[9] Streelman J.T., Alfaro M., Westneat M.W., Bellwood D.R., Karl S.A. (2002). Evolutionary History of the Parrotfishes: Biogeography, Ecomorphology, and Comparative Diversity. Evol. Int. J. Org. Evol..

[10] Nanami A. (2016). Parrotfish Grazing Ability: Interspecific Differences in Relation to Jaw-Lever Mechanics and Relative Weight of Adductor Mandibulae on an Okinawan Coral Reef. PeerJ.

[11] Pereira P.H.C., Santos M., Lippi D.L., Silva P. (2016). Ontogenetic Foraging Activity and Feeding Selectivity of the Brazilian Endemic Parrotfish *Scarus zelindae*. PeerJ.

[12] Carr A., Kemp A., Tibbetts I., Truss R., Drennan J. (2006). Microstructure of Pharyngeal Tooth Enameloid in the Parrotfish *Scarus rivulatus* (Pisces: Scaridae). J. Microsc..

[13] Viviani J., LeBlanc A., Rurua V., Mou T., Liao V., Lecchini D., Galzin R., Viriot L. (2022). Plicidentine in the Oral Fangs of Parrotfish (Scarinae, Labriformes). J. Anat..

[14] Goldberg E.G., Raab T.K., Desalles P., Briggs A.A., Dunbar R.B., Millero F.J., Woosley R.J., Young H.S., Micheli F., Mccauley D.J. (2019). Chemistry of the Consumption and Excretion of the Bumphead Parrotfish (*Bolbometopon muricatum*), a Coral Reef Mega-Consumer. Coral Reefs.

[15] Abdel-Aziz E.-S.H., Bawazeer F.A., El-Sayed Ali T., Al-Otaibi M. (2012). Sexual Patterns and Protogynous Sex Reversal in the Rusty Parrotfish, *Scarus ferrugineus* (Scaridae): Histological and Physiological Studies. Fish Physiol. Biochem..

[16] Godwin J., Sawby R., Warner R.R., Crews D., Grober M.S. (2000). Hypothalamic Arginine Vasotocin mRNA Abundance Variation across Sexes and with Sex Change in a Coral Reef Fish. Brain. Behav. Evol..

[17] Andradi-Brown D.A., Gress E., Wright G., Exton D.A., Rogers A.D. (2016). Reef Fish Community Biomass and Trophic Structure Changes across Shallow to Upper-Mesophotic Reefs in the Mesoamerican Barrier Reef, Caribbean. PLoS ONE.

[18] Nanami A. (2021). Spatial Distribution of Parrotfishes and Groupers in an Okinawan Coral Reef: Size-Related Associations in Relation to Habitat Characteristics. PeerJ.

[19] Guo W., Bokade R., Cohen A.L., Mollica N.R., Leung M., Brainard R.E. (2020). Ocean Acidification Has Impacted Coral Growth on the Great Barrier Reef. Geophys. Res. Lett..

[20] Burkepile D.E., Hay M.E. (2010). Impact of Herbivore Identity on Algal Succession and Coral Growth on a Caribbean Reef. PLoS ONE.

[21] Bezerra I.M., Gramacho K.P., Barreto M.A., Hackradt C.W., Leão Feitosa J.L., Torres R.A., Ferreira B.P., González-Wanguemert M., Félix-Hackradt F.C. (2018). Genetic Diversity and Gene Flow of the Threatened Brazilian Endemic Parrotfish *Scarus trispinosus* (Valenciennes, 1840). Mar. Environ. Res..

[22] Labrador K., Fortaleza M., Cabasan J., Elumba M., Nañola C. (2022). Genetic Diversity and Population Connectivity of the Greenblotch Parrotfish (*Scarus quoyi*, Valenciennes, 1840) within Southern Mindanao Inferred from Mitochondrial 16S rRNA. Philipp. J. Sci..

[23] Pereira P.H.C., Araujo J.C., Lima G.V., Côrtes L.G.F., Gomes E., Magris R.A. (2022). Effectiveness of Management Zones for Recovering Parrotfish Species within the Largest Coastal Marine Protected Area in Brazil. Sci. Rep..

[24] Smith L.L., Fessler J.L., Alfaro M.E., Streelman J.T., Westneat M.W. (2008). Phylogenetic Relationships and the Evolution of Regulatory Gene Sequences in the Parrotfishes. Mol. Phylogenet. Evol..

[25] Austin C.M., Tan M.H., Harrisson K.A., Lee Y.P., Croft L.J., Sunnucks P., Pavlova A., Gan H.M. (2017). De Novo Genome Assembly and Annotation of Australia’s Largest Freshwater Fish, the Murray Cod (*Maccullochella peelii*), from Illumina and Nanopore Sequencing Read. GigaScience.

[26] Fernandez-Silva I., Henderson J.B., Rocha L.A., Simison W.B. (2018). Whole-Genome Assembly of the Coral Reef Pearlscale Pygmy Angelfish (*Centropyge vrolikii*). Sci. Rep..

[27] Paris J.R., Stevens J.R., Catchen J.M. (2017). Lost in Parameter Space: A Road Map for Stacks. Methods Ecol. Evol..

[28] Galla S.J., Forsdick N.J., Brown L., Hoeppner M.P., Knapp M., Maloney R.F., Moraga R., Santure A.W., Steeves T.E. (2019). Reference Genomes from Distantly Related Species Can Be Used for Discovery of Single Nucleotide Polymorphisms to Inform Conservation Management. Genes.

[29] Bariche M., Bernardi G. (2009). Lack of a Genetic Bottleneck in a Recent Lessepsian Bioinvader, the Blue-Barred Parrotfish, *Scarus ghobban*. Mol. Phylogenet. Evol..

[30] Jawad L.A. (2018). A Comparative Morphological Investigation of Otoliths of Six Parrotfish Species (Scaridae) from the Solomon Islands. J. Fish Biol..

[31] Guo L., Zhang N., Zhu K.-C., Guo H.-Y., Liu B.-S., Zhang D.-C. (2019). The Complete Mitochondrial Genome of *Cheilinus oxycephalus* (Perciformes: Labridae). Mitochondrial DNA Part B Resour..

[32] Wingett S.W., Andrews S. (2018). FastQ Screen: A Tool for Multi-Genome Mapping and Quality Control. F1000Research.

[33] Xiao Y., Xiao Z., Ma D., Liu J., Li J. (2019). Genome Sequence of the Barred Knifejaw *Oplegnathus fasciatus* (Temminck & Schlegel, 1844): The First Chromosome-Level Draft Genome in the Family Oplegnathidae. GigaScience.

[34] Chen Y., Chen Y., Shi C., Huang Z., Zhang Y., Li S., Li Y., Ye J., Yu C., Li Z. (2018). SOAPnuke: A MapReduce Acceleration-Supported Software for Integrated Quality Control and Preprocessing of High-Throughput Sequencing Data. GigaScience.

[35] Liu B., Shi Y., Yuan J., Hu X., Zhang H., Li N., Li Z., Chen Y., Mu D., Fan W. (2020). Estimation of Genomic Characteristics by Analyzing K-Mer Frequency in de Novo Genome Projects. Quant. Biol..

[36] Hu J., Fan J., Sun Z., Liu S. (2020). NextPolish: A Fast and Efficient Genome Polishing Tool for Long-Read Assembly. Bioinformatics.

[37] Li H. (2013). Aligning Sequence Reads, Clone Sequences and Assembly Contigs with BWA-MEM. Quant. Biol..

[38] Manni M., Berkeley M.R., Seppey M., Simão F.A., Zdobnov E.M. (2021). BUSCO Update: Novel and Streamlined Workflows along with Broader and Deeper Phylogenetic Coverage for Scoring of Eukaryotic, Prokaryotic, and Viral Genomes. Mol. Biol. Evol..

[39] Flynn J.M., Hubley R., Goubert C., Rosen J., Clark A.G., Feschotte C., Smit A.F. (2020). RepeatModeler2 for Automated Genomic Discovery of Transposable Element Families. Proc. Natl. Acad. Sci. USA.

[40] Tarailo-Graovac M., Chen N. (2009). Using RepeatMasker to Identify Repetitive Elements in Genomic Sequences. Curr. Protoc. Bioinform..

[41] Stanke M., Keller O., Gunduz I., Hayes A., Waack S., Morgenstern B. (2006). AUGUSTUS: Ab Initio Prediction of Alternative Transcripts. Nucleic Acids Res..

[42] Haas B.J., Salzberg S.L., Zhu W., Pertea M., Allen J.E., Orvis J., White O., Buell C.R., Wortman J.R. (2008). Automated Eukaryotic Gene Structure Annotation Using EVidenceModeler and the Program to Assemble Spliced Alignments. Genome Biol..

[43] Birney E., Clamp M., Durbin R. (2004). GeneWise and Genomewise. Genome Res..

[44] Zhao N., Guo H.-B., Jia L., Deng Q.-X., Zhu C.-H., Zhang B. (2021). High-Quality Chromosome-Level Genome Assembly of Redlip Mullet (*Planiliza haematocheila*). Zool. Res..

[45] UniProt Consortium (2009). The Universal Protein Resource (UniProt) 2009. Nucleic Acids Res..

[46] Kanehisa M., Goto S. (2000). KEGG: Kyoto Encyclopedia of Genes and Genomes. Nucleic Acids Res..

[47] Zdobnov E.M., Apweiler R. (2001). InterProScan—An Integration Platform for the Signature-Recognition Methods in InterPro. Bioinformatics.

[48] Nawrocki E.P. (2014). Annotating Functional RNAs in Genomes Using Infernal. Methods Mol. Biol..

[49] Xu Z., Wang H. (2007). LTR_FINDER: An Efficient Tool for the Prediction of Full-Length LTR Retrotransposons. Nucleic Acids Res..

[50] Benson G. (1999). Tandem Repeats Finder: A Program to Analyze DNA Sequences. Nucleic Acids Res..

[51] Chalopin D., Naville M., Plard F., Galiana D., Volff J.-N. (2015). Comparative Analysis of Transposable Elements Highlights Mobilome Diversity and Evolution in Vertebrates. Genome Biol. Evol..

[52] Conte M.A., Gammerdinger W.J., Bartie K.L., Penman D.J., Kocher T.D. (2017). A High Quality Assembly of the Nile Tilapia (*Oreochromis niloticus*) Genome Reveals the Structure of Two Sex Determination Regions. BMC Genom..

[53] Gao B., Shen D., Xue S., Chen C., Cui H., Song C. (2016). The Contribution of Transposable Elements to Size Variations between Four Teleost Genomes. Mob. DNA.

[54] Ryu T., Herrera M., Moore B., Izumiyama M., Kawai E., Laudet V., Ravasi T. (2022). A Chromosome-Scale Genome Assembly of the False Clownfish, *Amphiprion ocellaris*. G3.

[55] Shao F., Han M., Peng Z. (2019). Evolution and Diversity of Transposable Elements in Fish Genomes. Sci. Rep..

[56] Mather N., Traves S.M., Ho S.Y.W. (2020). A Practical Introduction to Sequentially Markovian Coalescent Methods for Estimating Demographic History from Genomic Data. Ecol. Evol..

[57] Danecek P., Bonfield J.K., Liddle J., Marshall J., Ohan V., Pollard M.O., Whitwham A., Keane T., McCarthy S.A., Davies R.M. (2021). Twelve Years of SAMtools and BCFtools. GigaScience.

[58] Jones D.D., Rivera Hernández J.M., Shervette V.R. (2021). Princess Parrotfish Scarus Taeniopterus Age, Growth, Maturity, and Transition. Environ. Biol. Fishes.

[59] Emms D.M., Kelly S. (2019). OrthoFinder: Phylogenetic Orthology Inference for Comparative Genomics. Genome Biol..

[60] Katoh K., Standley D.M. (2013). MAFFT Multiple Sequence Alignment Software Version 7: Improvements in Performance and Usability. Mol. Biol. Evol..

[61] Stamatakis A. (2014). RAxML Version 8: A Tool for Phylogenetic Analysis and Post-Analysis of Large Phylogenies. Bioinformatics.

[62] Yang Z. (2007). PAML 4: Phylogenetic Analysis by Maximum Likelihood. Mol. Biol. Evol..

[63] Mendes F.K., Vanderpool D., Fulton B., Hahn M.W. (2021). CAFE 5 Models Variation in Evolutionary Rates among Gene Families. Bioinformatics.

[64] Yu G., Wang L.-G., Han Y., He Q.-Y. (2012). clusterProfiler: An R Package for Comparing Biological Themes among Gene Clusters. Omics J. Integr. Biol..

[65] Gao F., Chen C., Arab D.A., Du Z., He Y., Ho S.Y.W. (2019). EasyCodeML: A Visual Tool for Analysis of Selection Using CodeML. Ecol. Evol..

[66] Zeisel A., Zuk O., Domany E. (2011). FDR Control with Adaptive Procedures and FDR Monotonicity. Ann. Appl. Stat..

[67] Cantalapiedra C.P., Hernández-Plaza A., Letunic I., Bork P., Huerta-Cepas J. (2021). eggNOG-Mapper v2: Functional Annotation, Orthology Assignments, and Domain Prediction at the Metagenomic Scale. Mol. Biol. Evol..

[68] McCartney A.M., Hilario E., Choi S.-S., Guhlin J., Prebble J.M., Houliston G., Buckley T.R., Chagné D. (2021). An Exploration of Assembly Strategies and Quality Metrics on the Accuracy of the Rewarewa (*Knightia excelsa*) Genome. Mol. Ecol. Resour..

[69] Sims D., Sudbery I., Ilott N.E., Heger A., Ponting C.P. (2014). Sequencing Depth and Coverage: Key Considerations in Genomic Analyses. Nat. Rev. Genet..

[70] Liu D., Wang X., Guo H., Zhang X., Zhang M., Tang W. (2021). Chromosome-Level Genome Assembly of the Endangered Humphead Wrasse *Cheilinus undulatus*: Insight into the Expansion of Opsin Genes in Fishes. Mol. Ecol. Resour..

[71] Tshilate T.S., Ishengoma E., Rhode C. (2023). A First Annotated Genome Sequence for Haliotis Midae with Genomic Insights into Abalone Evolution and Traits of Economic Importance. Mar. Genom..

[72] Xu P., Zhang X., Wang X., Li J., Liu G., Kuang Y., Xu J., Zheng X., Ren L., Wang G. (2014). Genome Sequence and Genetic Diversity of the Common Carp, *Cyprinus carpio*. Nat. Genet..

[73] Mattingsdal M., Jentoft S., Tørresen O.K., Knutsen H., Hansen M.M., Robalo J.I., Zagrodzka Z., André C., Gonzalez E.B. (2018). A Continuous Genome Assembly of the Corkwing Wrasse (*Symphodus melops*). Genomics.

[74] Bernardi G., DeBiasse M., Escalona M., Marimuthu M.P.A., Nguyen O., Sacco S., Beraut E., Miller C., Toffelmier E., Shaffer H.B. (2022). Reference Genome of the California Sheephead, *Semicossyphus pulcher* (Labridae, Perciformes), A Keystone Fish Predator in Kelp Forest Ecosystems. J. Hered..

[75] Kang J., Ramirez-Calero S., Paula J.R., Chen Y., Schunter C. (2023). Gene Losses, Parallel Evolution and Heightened Expression Confer Adaptations to Dedicated Cleaning Behaviour. BMC Biol..

[76] Symonová R., Suh A. (2019). Nucleotide Composition of Transposable Elements Likely Contributes to AT/GC Compositional Homogeneity of Teleost Fish Genomes. Mob. DNA.

[77] Mäkinen V., Salmela L., Ylinen J. (2012). Normalized N50 Assembly Metric Using Gap-Restricted Co-Linear Chaining. BMC Bioinform..

[78] Zhang H.-H., Xu M.-R.-X., Wang P.-L., Zhu Z.-G., Nie C.-F., Xiong X.-M., Wang L., Xie Z.-Z., Wen X., Zeng Q.-X. (2020). High-Quality Genome Assembly and Transcriptome of *Ancherythroculter nigrocauda*, an Endemic Chinese Cyprinid Species. Mol. Ecol. Resour..

[79] Kidwell M.G. (2002). Transposable Elements and the Evolution of Genome Size in Eukaryotes. Genetica.

[80] Kushwaha B., Nagpure N.S., Srivastava S., Pandey M., Kumar R., Raizada S., Agarwal S., Singh M., Basheer V.S., Kumar R.G. (2023). Genome Size Estimation and Its Associations with Body Length, Chromosome Number and Evolution in Teleost Fishes. Gene.

[81] Canapa A., Barucca M., Biscotti M.A., Forconi M., Olmo E. (2015). Transposons, Genome Size, and Evolutionary Insights in Animals. Cytogenet. Genome Res..

[82] Frantz L.A.F., Mullin V.E., Pionnier-Capitan M., Lebrasseur O., Ollivier M., Perri A., Linderholm A., Mattiangeli V., Teasdale M.D., Dimopoulos E.A. (2016). Genomic and Archaeological Evidence Suggest a Dual Origin of Domestic Dogs. Science.

[83] Johnson R.N., O’Meally D., Chen Z., Etherington G.J., Ho S.Y.W., Nash W.J., Grueber C.E., Cheng Y., Whittington C.M., Dennison S. (2018). Adaptation and Conservation Insights from the Koala Genome. Nat. Genet..

[84] Miller W., Schuster S.C., Welch A.J., Ratan A., Bedoya-Reina O.C., Zhao F., Kim H.L., Burhans R.C., Drautz D.I., Wittekindt N.E. (2012). Polar and Brown Bear Genomes Reveal Ancient Admixture and Demographic Footprints of Past Climate Change. Proc. Natl. Acad. Sci. USA.

[85] Nadachowska-Brzyska K., Burri R., Olason P.I., Kawakami T., Smeds L., Ellegren H. (2013). Demographic Divergence History of Pied Flycatcher and Collared Flycatcher Inferred from Whole-Genome Re-Sequencing Data. PLoS Genet..

[86] Lu L., Zhao J., Li C. (2020). High-Quality Genome Assembly and Annotation of the Big-Eye Mandarin Fish (*Siniperca knerii*). G3 GenesGenomesGenetics.

[87] Barash M.S. (2011). Environmental Changes in the Neogene and the Biotic Response. Oceanology.

[88] Arias C.F., Dikow R.B., McMillan W.O., De León L.F. (2021). De Novo Genome Assembly of the Electric Fish Brachyhypopomus Occidentalis (Hypopomidae, Gymnotiformes). Genome Biol. Evol..

[89] Hayward B., Kawagata S., Grenfell H., Sabaa A., O’Neill T. (2007). Last Global Extinction in the Deep Sea during the Mid-Pleistocene Climate Transition. Paleoceanography.

[90] Modys A.B., Oleinik A., Mortlock R.A., Toth L.T., Precht W.F. (2022). Climate-Modulated Range Expansion of Reef-Building Coral Communities off Southeast Florida during the Late Holocene. Front. Mar. Sci..

[91] Li J., Bian C., Yi Y., Yu H., You X., Shi Q. (2021). Temporal Dynamics of Teleost Populations during the Pleistocene: A Report from Publicly Available Genome Data. BMC Genom..

[92] Zhou S.Z., Jijun L., Zhang S.Q., Zhao J.D., Cui J.X., Ehlers J., Gibbard P.L. (2004). Quaternary Glaciations in China. Developments in Quaternary Sciences.

[93] Westneat M.W., Alfaro M.E., Wainwright P.C., Bellwood D.R., Grubich J.R., Fessler J.L., Clements K.D., Smith L.L. (2005). Local Phylogenetic Divergence and Global Evolutionary Convergence of Skull Function in Reef Fishes of the Family Labridae. Proc. Biol. Sci..

[94] Westneat M.W., Alfaro M.E. (2005). Phylogenetic Relationships and Evolutionary History of the Reef Fish Family Labridae. Mol. Phylogenet. Evol..

[95] Hughes L.C., Nash C.M., White W.T., Westneat M.W. (2023). Concordance and Discordance in the Phylogenomics of the Wrasses and Parrotfishes (Teleostei: Labridae). Syst. Biol..

[96] Roff G., Ledlie M.H., Ortiz J.C., Mumby P.J. (2011). Spatial Patterns of Parrotfish Corallivory in the Caribbean: The Importance of Coral Taxa, Density and Size. PLoS ONE.

[97] Denny C., Schiel D. (2001). Feeding Ecology of the Banded Wrasse *Notolabrus fucicola* (Labridae) in Southern New Zealand. N. Z. J. Mar. Freshw. Res..

[98] Greenwood P.H., Rosen D.E., Weitzman S.H., Myers G.S. (1966). George S. Phyletic Studies of Teleostean Fishes, with a Provisional Classification of Living Forms. Bull. AMNH.

[99] Black A., Imhoff V., Leese J., Weimann S., Gumm J., Richter M., Itzkowitz M. (2014). Attack Intensity by Two Species of Territorial Damselfish (Pomacentridae) as Estimates of Competitive Overlap with Two Species of Wrasse (Labridae). J. Ethol..

[100] Mikami Y. (2013). Phylogenic Relationship of Labridae Species Deduced from Comparative Dissection. Anat. Rec..

[101] Liang Y., Pan J.-M., Zhu K.-C., Xian L., Guo H.-Y., Liu B.-S., Zhang N., Yang J.-W., Zhang D.-C. (2023). Genome-Wide Identification of Trachinotus Ovatus Antimicrobial Peptides and Their Immune Response against Two Pathogen Challenges. Mar. Drugs.

[102] Parkin J., Cohen B. (2001). An Overview of the Immune System. Lancet.

[103] Arai T., Amalina R., Bachok Z. (2015). Fatty Acid Composition Indicating Diverse Habitat Use in Coral Reef Fishes in the Malaysian South China Sea. Biol. Res..

[104] Sukumaran S., Sebastian W., Gopalakrishnan A., Mathew O.K., Vysakh V.G., Rohit P., Jena J.K. (2023). The Sequence and de Novo Assembly of the Genome of the Indian Oil Sardine, *Sardinella longiceps*. Sci. Data.

[105] Gerlach G., Atema J., Kingsford M.J., Black K.P., Miller-Sims V. (2007). Smelling Home Can Prevent Dispersal of Reef Fish Larvae. Proc. Natl. Acad. Sci. USA.

[106] Zhang S., Song Y., Liu M., Yuan Z., Zhang M., Zhang H., Seim I., Fan G., Liu S., Liu X. (2023). Chromosome-Level Genome of Butterflyfish Unveils Genomic Features of Unique Colour Patterns and Morphological Traits. DNA Res. Int. J. Rapid Publ. Rep. Genes Genomes.

[107] He S., Li L., Lv L.-Y., Cai W.-J., Dou Y.-Q., Li J., Tang S.-L., Chen X., Zhang Z., Xu J. (2020). Mandarin Fish (Sinipercidae) Genomes Provide Insights into Innate Predatory Feeding. Commun. Biol..

[108] Bao L., Tian C., Liu S., Zhang Y., Elaswad A., Yuan Z., Khalil K., Sun F., Yang Y., Zhou T. (2019). The Y Chromosome Sequence of the Channel Catfish Suggests Novel Sex Determination Mechanisms in Teleost Fish. BMC Biol..

[109] Todd E.V., Ortega-Recalde O., Liu H., Lamm M.S., Rutherford K.M., Cross H., Black M.A., Kardailsky O., Marshall Graves J.A., Hore T.A. (2019). Stress, Novel Sex Genes, and Epigenetic Reprogramming Orchestrate Socially Controlled Sex Change. Sci. Adv..

